# Assessing the use of the novel tool Claude 3 in comparison to ChatGPT 4.0 as an artificial intelligence tool in the diagnosis and therapy of primary head and neck cancer cases

**DOI:** 10.1007/s00405-024-08828-1

**Published:** 2024-08-07

**Authors:** Benedikt Schmidl, Tobias Hütten, Steffi Pigorsch, Fabian Stögbauer, Cosima C. Hoch, Timon Hussain, Barbara Wollenberg, Markus Wirth

**Affiliations:** 1 Department of Otolaryngology Head and Neck Surgery, Technical University Munich, Munich, Germany; 2 Department of RadioOncology, Technical University Munich, Munich, Germany; 3 Institute of Pathology, Technical University Munich, Munich, Germany

**Keywords:** Claude 3 Opus, HNSCC, Multidisciplinary Tumorboard, Artificial Intelligence, LLM

## Abstract

Head and neck squamous cell carcinoma (HNSCC) is a complex malignancy that requires a multidisciplinary tumor board approach for individual treatment planning. In recent years, artificial intelligence tools have emerged to assist healthcare professionals in making informed treatment decisions. This study investigates the application of the newly published LLM Claude 3 Opus compared to the currently most advanced LLM ChatGPT 4.0 for the diagnosis and therapy planning of primary HNSCC. The results were compared to that of a conventional multidisciplinary tumor board; (2) Materials and Methods: We conducted a study in March 2024 on 50 consecutive primary head and neck cancer cases. The diagnostics and MDT recommendations were compared to the Claude 3 Opus and ChatGPT 4.0 recommendations for each patient and rated by two independent reviewers for the following parameters: clinical recommendation, explanation, and summarization in addition to the Artificial Intelligence Performance Instrument (AIPI); (3) Results: In this study, Claude 3 achieved better scores for the diagnostic workup of patients than ChatGPT 4.0 and provided treatment recommendations involving surgery, chemotherapy, and radiation therapy. In terms of clinical recommendations, explanation and summarization Claude 3 scored similar to ChatGPT 4.0, listing treatment recommendations which were congruent with the MDT, but failed to cite the source of the information; (4) Conclusion: This study is the first analysis of Claude 3 for primary head and neck cancer cases and demonstrates a superior performance in the diagnosis of HNSCC than ChatGPT 4.0 and similar results for therapy recommendations. This marks the advent of a newly launched advanced AI model that may be superior to ChatGPT 4.0 for the assessment of primary head and neck cancer cases and may assist in the clinical diagnostic and MDT setting.

## Highlights

Claude 3 Opus

HNSCC

Multidisciplinary Tumorboard

Artificial Intelligence

LLM

## Introduction

The advent of artificial intelligence (AI), especially deep learning (DL) and natural language processing (NLP), has given rise to Large Language Models (LLMs) like Generative Pre-trained Transformer (GPT) [1, 2]. Many publications have evaluated the use of ChatGPT in clinical decision making so far with mixed results [3–6]. Some previous studies assessed the potential benefit of an AI-assisted MDT and showed high concordance of ChatGPT 4.0-generated results with non-AI-assisted MTB recommendations. Still, several limitations including a lack of solid oncological training and validation, and a lack of tailoring the treatment to the patient’s specific needs [1, 7, 8] remain. While ChatGPT 4.0 surpassed ChatGPT 3.5 in most studies and is currently considered the most advanced LLM, a new LLM was introduced in March 2024, Claude 3 Opus, which outperforms ChatGPT 4.0 in most of the common evaluation benchmarks for AI systems, including undergraduate level expert knowledge (MMLU), graduate level expert reasoning (GPQA), basic mathematics (GSM8K), and is therefore considered as a worthy successor for operational and standard use cases of LLMs [1, 8, 9].

In prior publications Claude 2 already surpassed ChatGPT 3.5 in answering questionnaires [10], but there are currently no publications yet regarding the use of Claude 3 Opus on PubMed.

In terms of cancer treatment decision making in a multidisciplinary tumor board (MDT) setting, head and neck squamous cell carcinomas are particularly challenging. HNSCC is one of the most prevalent cancer types originating from a complex anatomical region. and has molecular heterogeneous character and a rising incidence of HPV-positive cases [11, 12]. MDTs aim to optimize patient outcomes through an individualized and collaborative approach of different healthcare professionals [7, 13], including medical and surgical oncologists, radiation oncologists, pathologists, and radiologists [7, 11]. One of the main advantages of an MDT-approach is the ability to customize treatment plans to individual patients’ needs, as seen especially in molecular tumor boards for the most challenging cases [7, 14]. Nevertheless, MDTs are oftentimes limited by geographic barriers, costs, and treatment delays.

LLMs have the advantage of accessing large datasets in a short amount of time. The relevant information of recent studies, but also historical data may be gathered and summarized by an LLM as the basis of a modern approach to discuss oncological cases [7, 13, 14]. This ability to organize and structure data is the main reason LLMs are investigated as a tool to assist, or even guide MDT-based decision making in the future [3, 15].

This study is the first to evaluate the use of Claude 3 Opus in the clinical diagnostic setting and as a tool assisting the MDT for primary head and neck cancer treatment decision making, comparing its answers to those of the currently most advanced LLM, ChatGPT 4.0. This study aims to assess the potential of Claude 3 Opus in providing recommendations for patients with primary head and neck cancer.

## Materials and methods

### Patient cohort

This study included patients with a verified head and neck cancer diagnosis and no evidence of distant metastasis. Cases involving recurrent situations were excluded from the study. The electronic patient file and MDT documents provided clinical and histological tumor characteristics and patient ages before treatment initiation. This study comprised a total of 50 patients, all of whom were discussed during the October 2023 MDT meeting at the Department of Otorhinolaryngology/Head and Neck Surgery, Klinikum rechts der Isar, Technical University of Munich. The patients’ ages spanned from 51 to 82 years. To ensure patient confidentiality, the data were anonymized before being shared with the researchers, making patient identification impossible. This study was approved by the ethics committee of the Technical University of Munich. The characteristics of the patient cohort are depicted in Table 1.

**Table 1:** Overview of the patient cohort and the information entered into Claude 3 Opus and ChatGPT 4.0. In addition, the result of the MDT presentation is also depicted in this table, even though Claude 3 and ChatGPT were not able to access this information. Abbreviations: NA = not available, DVT = deep vein thrombosis, aHTN = arterial hypertension, DM = diabetes mellitus, TLE = total laryngectomy, sND = selective neck dissection, adj = adjuvant, RCTx = radiochemotherapy, KPS = Karnofsky Performance Index, RTx = radiotherapy

ID	Age	cTx	cNx	cMx	Localization	p16 status	Smoking status	Comorbidities	KPS %	Recommendation of the MDT
1	74	4a	0	0	Transglottic larynx left	NA	No more, 25 py	aHTN	100	Surgery + Adj. RTx vs. RCTx
2	62	2	2b	0	Hypopharynx right	NA	Active, 40 py	pAVK, DM type II	60	Surgery + Adj. RCTx
3	60	3	0	0	Oropharynx right	-	Active, 25py	aHTN	100	Surgery + Adj. RTx
4	61	2	1	0	Hypopharynx left	NA	Rarely	Chronic renal insufficiency	50	Surgery + Adj. RTx
5	61	3	1	0	Oropharynx left	+	Never	aHTN	100	Surgery + Adj. RCTx
6	52	2	1	0	Oropharynx left	+	Never	aHTN	100	Surgery + Adj. RCTx
7	63	1b	0	0	Glottic larynx	NA	Active, 40 py	Alcohol abuse	80	Surgery + Clinical Controls
8	73	2	0	0	Oropharynx right	+	Never	DVT 2015	100	Surgery + Adj. RTx
9	70	3	0	0	Right nasal cavity	NA	Never	CLL, aHTN	100	Surgery + Adj. RTx
10	57	4a	1	0	Supraglottic larynx to subglottic with thoracic extension and into the trachea.	NA	Never	h.o. pneumonia, COPD	60	RCTx
11	74	1	1	0	Oropharynx right	+	Active, 20 py	aHTN	90	Surgery + Adj. RTx
12	61	4a	1	0	Glottic larynx	NA	Active, 20 py	aHTN, DM type II	70	Surgery + Adj. RTx
13	59	3	2	0	Hypopharynx right	NA	Active, 20 py	aHTN	100	Surgery + Adj. RTx
14	64	2	2a	0	Supraglottic larynx	NA	Active, 40 py	aHTN	100	Surgery + Adj. RTx
15	63	3	2c	0	Oropharynx right	-	Active, 10 py	DM type II	100	RCTx
16	82	4a	1	0	Glottic larynx	NA	Active, 20 py	aHTN, Chronic renal insufficiency	90	Surgery + Adj. RTx
17	71	1	1	0	Oropharynx left	-	No more, 30 py	aHTN	80	Surgery + Adj. RTx
18	64	2	0	0	Hypopharynx right	NA	Active, 60 py	None	100	Surgery + Adj. RTx
19	68	1a	0	0	Glottic larynx	NA	Active, 15 py	aHTN	100	Surgery
20	79	2	2c	0	Oropharynx right	+	Never	aHTN, DM type II	90	Surgery + Adj. RTx
21	71	2	2a	0	Hypopharynx left	NA	No more, 60 py	DM type II	100	Surgery + Adj. RTx
22	51	3	0	0	Supraglottic larynx	NA	Active, 30 py	aHTN	100	Surgery + Adj. RTx
23	77	2	1	0	Hypopharynx left	NA	No more, 25 py	None	90	Surgery + Adj. RTx
24	56	3	2c	0	Oropharynx right	-	Never	aHTN	90	RCTx
25	80	1	0	0	Oropharynx left	-	Active, 30 py	aHTN, Chronic renal insufficiency	90	Surgery
26	59	1b	0	0	Glottic larynx	NA	Active, 40 py	None	100	Surgery
27	66	2	2a	0	Hypopharynx right	NA	No more, 50 py	aHTN	100	Surgery + Adj. RTx
28	71	4a	2c	0	Glottic larynx	NA	Active, 50 py	aHTN, DM type II, Arrythmia	60	Surgery + Adj. RTx
29	73	2	2b	0	Oropharynx left	-	Active, 20 py	aHTN	90	Surgery + Adj. RTx
30	74	3	1	0	Hypopharynx right	NA	Active, 40 py	aHTN	80	RCTx
31	65	2	1	0	Oropharynx right	-	No more, 40 py	aHTN, DM type II	90	Surgery + Adj. RTx
32	69	3	2b	0	Glottic larynx	NA	Active, 10 py	aHTN	90	Surgery + Adj. RTx
33	70	2	2a	0	Hypopharynx right	NA	Active, 20 py	aHTN	100	Surgery + Adj. RTx
34	59	1a	0	0	Glottic larynx	NA	Never	None	100	Surgery
35	72	2	0	0	Oropharynx left	-	Active, 10 py	aHTN, DM type II	100	Surgery
36	75	4a	2c	0	Glottic larynx	NA	Active, 50 py	Chronic renal insufficiency, aHTN	60	RTx
37	80	4a	2a	0	Glottic larynx	NA	No more, 50 py	aHTN, DM type II, Heart insufficiency	70	Surgery + Adj. RCTx
38	66	3	2	0	Oropharynx left	+	Never	None	100	Surgery + Adj. RTx
39	76	3	1	0	Hypopharynx right	NA	Active, 30 py	DM type II	90	Surgery + Adj. RTx
40	71	1b	0	0	Glottic larynx	NA	Active, 50 py	aHTN, h.o. prostate cancer	80	Surgery
41	59	2	0	0	Oropharynx right	+	Never	None	100	Surgery
42	61	2	1	0	Hypopharynx right	NA	Active, 30 py	aHTN	100	Surgery + Adj. RTx
43	68	3	1	0	Hypopharynx left	NA	Active, 40 py	aHTN	80	Surgery + Adj. RTx
44	63	3	2c	0	Oropharynx left	-	Active, 60 py	aHTN, h.o. arrhythmia	80	Surgery + Adj. RTx
45	59	1a	1	0	Glottic larynx	NA	Never	None	100	Surgery + Adj. RTx
46	72	4a	2a	0	Glottic larynx	NA	Active, 80 py	DM type II, aHTN, h.o stroke, h.o. pneumonia	60	RCTx
47	70	2	0	0	Oropharynx left	-	Active, 50 py	aHTN	100	Surgery
48	64	3	1	0	Hypopharynx left	NA	No more, 20 py	aHTN	100	Surgery + Adj. RTx
49	77	2	1	0	Oropharynx left	+	Active, 20 py	aHTN, Chronic renal insufficiency	80	Surgery + Adj. RTx
50	68	2	2c	0	Hypopharynx left	NA	Active, 50 py	None	80	Surgery + Adj. RTx

### Artificial Intelligence/ Claude 3 Opus and ChatGPT and prompt format and data evaluation

Claude 3 Opus and ChatGPT are AI-powered chatbots that are accessible to the public. These chatbots utilize transformer-based language models to generate human-like text responses. Users can interact with these LLMs by submitting questions through a website interface. The LLMs then analyze the contextual relationships between the words in the user’s query to formulate a response. In this study, a standardized prompt format was employed to input patient information into Claude 3 Opus and ChatGPT, simulating the presentation of an individual patient case in multidisciplinary team (MDT) meetings. The prompt used in this study was designed to mirror the standard case presentation format used in MDTs, enabling a direct comparison between the chatbot’s recommendations and those of the MDT. For assessing the performance for the diagnostic workup of patients 4 different prompts were tested and evaluated to assess the influence of the design of the prompt. Additionally for the assessment of the therapy recommendations 8 different prompts were tested and the best scoring prompt was used for the subsequent analysis of the 50 cases. The prompt was as follows: “A (XX) year old patient with a cT (XX) cN (XX) squamous cell carcinoma of the (XX), the patient (XX) smokes, (XX) and has the following secondary diseases (XX) with a Karnofsky Index of (XX). The patient is presented in an interdisciplinary tumor board. What treatment options are available and which option do you think leads to the best prognosis?”. This study simulates the conversation between an oncologist or head and neck surgeon in the MDT. For instance, the following scenario could be presented: “A 74-year-old patient has been diagnosed with a cT4a cN0 squamous cell carcinoma of the left transglottic larynx. The patient is a former smoker with a history of 25 pack-years and suffers from arterial hypertension. The patient’s Karnofsky Index is 100%. The case is discussed in a pre-therapeutic interdisciplinary tumor board. What are the available treatment options, and which one do you believe offers the best prognosis?” No further interaction was initiated after each response; ChatGPT’s history was erased, and the next question was asked. The supplementary data contains the prompts used in the study. To prevent any influence from previous responses, a new session was initiated for each prompt. B.S. collected Claude 3 and ChatGPT’s responses, which were then independently evaluated by B.S. and T.H. The answers provided by Claude 3 Opus and ChatGPT 4.0 were assessed using the grading scales for Summarization, Clinical Recommendation, and Explanation, as employed by Sorin et al. 2023 [16] and a modified version of the Artificial Intelligence Performance Instrument (AIPI) as proposed by Lechien et al. [17]. For this, Claude 3 and ChatGPT were interrogated for the most likely diagnosis, differential diagnoses (What are your differential diagnoses? ), additional examinations (What are your additional examinations to find the diagnosis? ). The question for potential therapeutic approach(es) (What are your treatment(s) for the primary diagnosis? ) was not asked since the recommendations for therapy were already tested with the other evaluation tool. The entire treatment history was considered. Cohen’s kappa coefficient was used to measure inter-rater reliability.

## Results

**Fig. 1:**
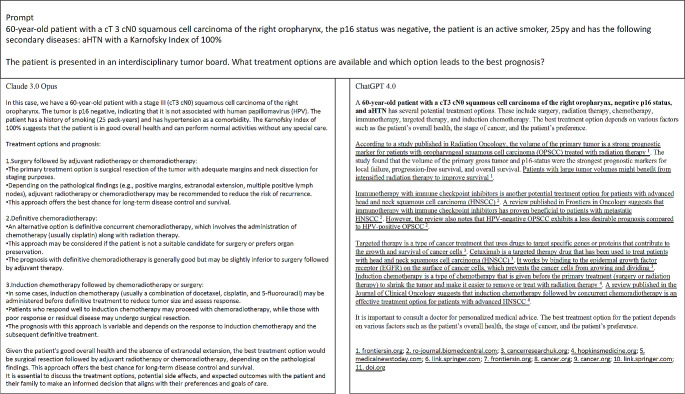
Depiction of an exemplary prompt and the corresponding responses by Claude 3 Opus and ChatGPT 4.0. The answers were copied from the response page

In the first part of the examination of Claude 3, the clinical consultation of ten consecutive primary HNSCC cases was evaluated, including the most likely diagnosis, differential diagnoses, and additional examinations. An exemplary prompt and the answers generated by Claude 3 Opus and ChatGPT 4.0 are depicted in Fig. 1.

**Fig. 2:**
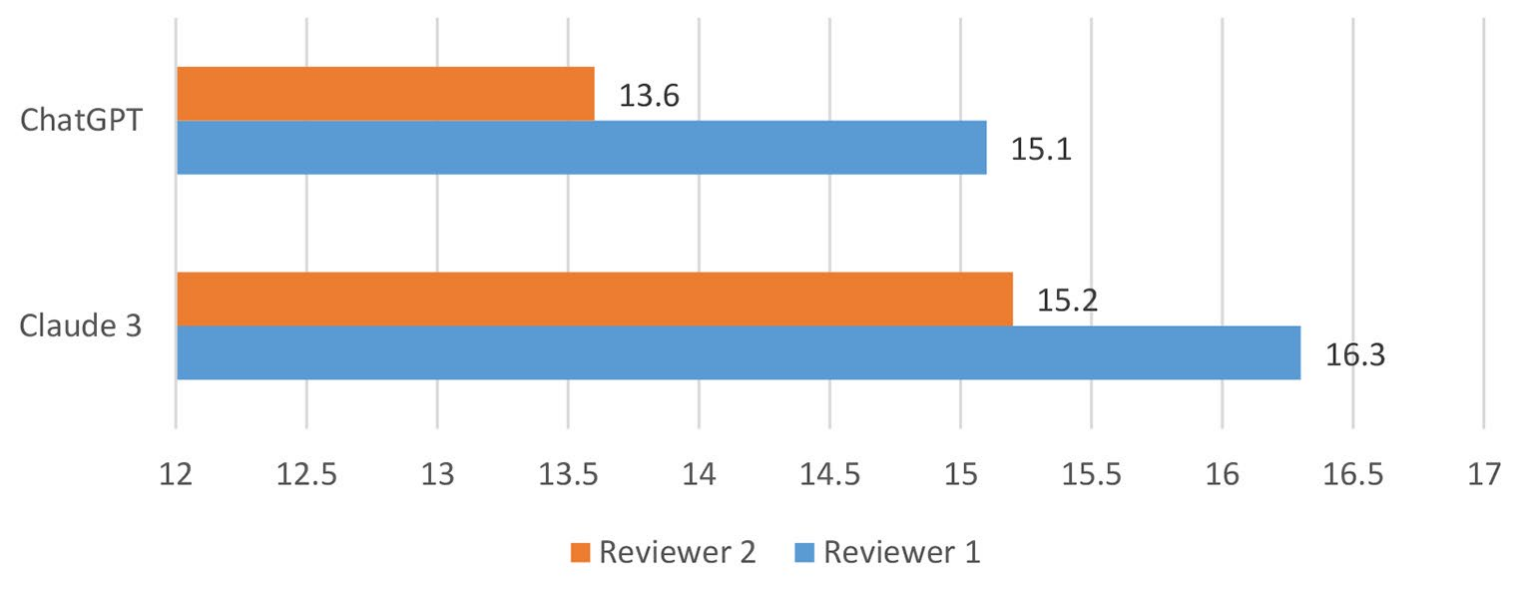
Rating of the overall performance of Claude 3 Opus and ChatGPT 4.0 for the diagnosis of primary HNSCC using the modified Artificial Intelligence Performance Index (AIPI). The rating was performed by two independent reviewers. The maximum score of the modified AIPI was 17 since the therapy options were not scored in the modified version of the AIPI

Claude 3 Opus answered the prompts in a fast way and achieved high scores in the Artificial Intelligence Performance Instrument (AIPI). Claude 3 surpassed ChatGPT 4.0 in terms of overall score in this evaluation tool (Fig. 2).

When analyzing the answers of the AIPI separately for ChatGPT 4.0 and Claude 3 Opus, there are significant discrepancies, as seen for example in answer 5 regarding the primary diagnosis, where ChatGPT 4.0 listed oropharyngeal dysphagia as the most likely diagnosis instead of a primary malignancy. Whereas question 6 was answered by ChatGPT and Claude 3 in a similar way and reached a similar score of the AIPI, other questions reached higher scores for Claude 3 Opus. Question 1 of the AIPI also had a large discrepancy. Claude 3 Opus reached high scores for considering the medical and surgical history of patients (Question 1), and also for diagnosis planning (Question 6) and additional examinations (Question 7). Claude also demonstrated to be superior in terms of prioritizing the most relevant diagnostic examinations (Question 8).

**Fig. 3:**
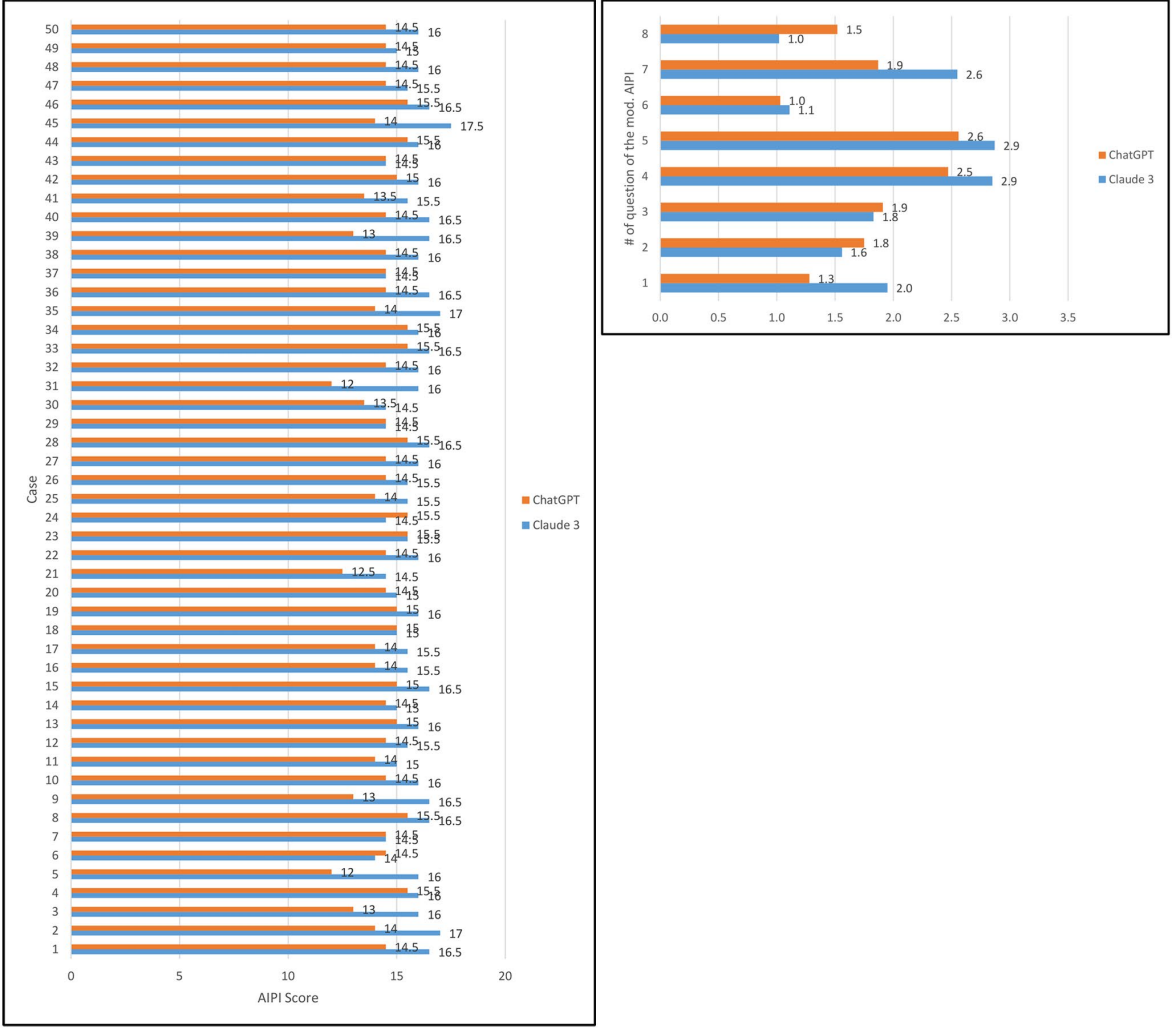
Rating of the performance of Claude 3 Opus and ChatGPT 4.0 for the diagnosis of primary HNSCC using the modified Artificial Intelligence Performance Index (AIPI). (**A**) Overall performance. (**B**) Performance for each case. (**C**) Performance for each question of the AIPI. The maximum score of the modified AIPI was 17 since the therapy options were not scored in the modified version of the AIPI

Claude 3 Opus suggested EBV and HPV analysis for the majority of cases, even for laryngeal cancer, where no statistical association is seen, and testing is not part of the routine diagnostic work up. When analyzing the results of different cases one sees larger differences between Claude 3 and ChatGPT for the majority of cases, especially in cases involving oropharyngeal squamous cell carcinoma (Fig. 3). The design of the prompt influences ChatGPT and Claude 3, as seen in the analysis of 8 different prompts for the recommendation of the best therapy. These prompts and the answers by Claude 3 Opus and ChatGPT 4.0 are depicted in the Supplementary.

**Fig. 4:**
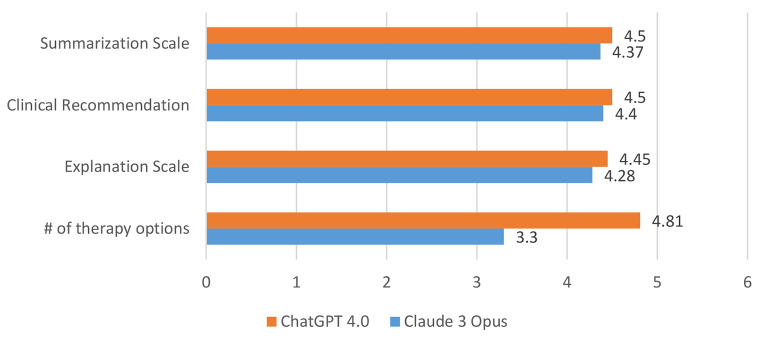
Rating of the performance of Claude 3 Opus and ChatGPT 4.0 by comparison of the number of treatment options per case and the grading of summarization of text, clinical recommendation, and explanation on the decision made by two independent reviewers. Each bar is the average of the two independent reviewers grading. # = number

Additionally, the recommendations for primary HNSCC by Claude 3 Opus were tested. The LLM answered the prompts of the MDT in a fast way and listed the various treatment options. The most common primary treatment modalities available for potential use in a primary or adjuvant setting, including surgery, radiotherapy, and chemotherapy were discussed by the LLM. When comparing the MDT recommendations with those of Claude 3 Opus, two independent reviewers reached an agreement measured by Cohen’s κ of 0.287 for summarization of text, of 0.324 for clinical recommendation, and 0.08 for explanation on the decision made. ChatGPT 4.0 reached a Cohen’s κ of 0.44 for summarization of text, of 0.159 for clinical recommendation, and 0.306 for explanation on the decision made (Supplementary Table). Overall, Claude 3 suggested a lower number of therapy options (3.3 vs. 4.81) and reached similar scores for the scales of clinical recommendation, explanation, and summarization (Fig. 4).

**Fig. 5:**
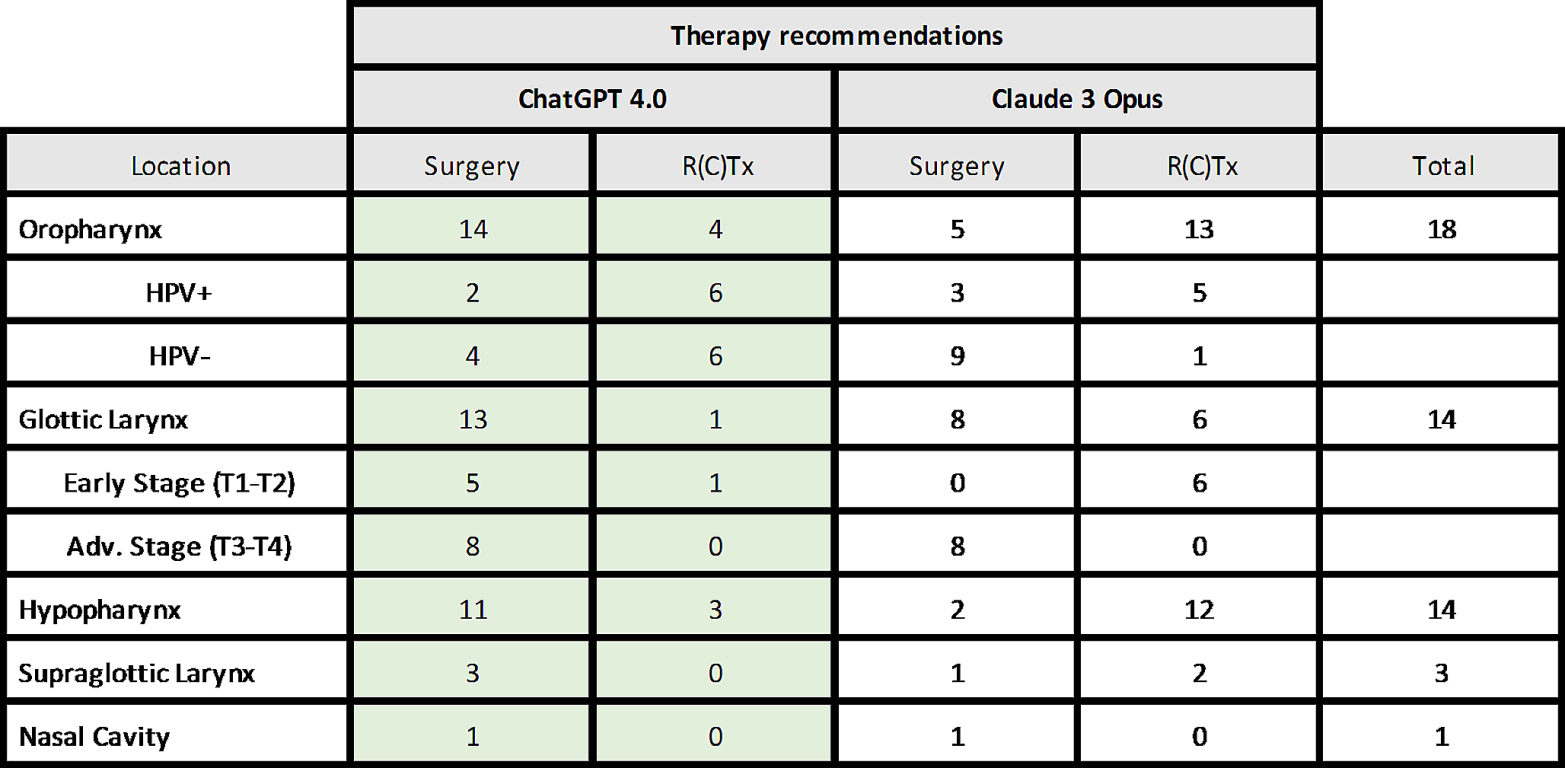
Overview of the recommended therapy and differences between ChatGPT 4.0 and Claude 3 Opus for different anatomical subsites. # = number, Adv = advanced

Compared to ChatGPT 4.0, immunotherapy was never a therapy option of Claude 3 for the patients in this study of primary HNSCC as a therapy option, while ChatGPT 4.0 recommended immunotherapy for patients without regard to the PD-L1 status or combined positive score (CPS). On the other hand, Claude 3 Opus recommended radiochemotherapy (RCTx) or induction chemotherapy (using docetaxel, cisplatin, and 5-fluorouracil) for tumor downsizing or organ preservation in many cases. When one compares the preferred therapy, there is a heterogeneous result depending on the anatomical subsite. The subsite analysis results in small numbers for each group but led to ChatGPT recommending surgery for 72.2% (13 out of 18) of OPSCC and Claude 3 Opus recommending surgery for 27.8% of OPSCC (5 out of 18).

Further analysis of HPV-positive versus HPV-negative specimen resulted in the recommendation of surgery for 25.0% of HPV-associated OPSCC (2 out of 8) by ChatGTP and 37.5% (3 out of 8) by Claude 3 Opus. For HPV-negative cases, ChatGPT recommended surgery for 40.0% (4 out of 10) and Claude 3 Opus recommended surgery for 90% (9 out of 10) of the patients. For glottic laryngeal cancer ChatGPT recommended surgery for most of the cases (92.9%), with a special emphasis on advanced cases. Claude 3 Opus on the other hand recommended radiotherapy for 42.3% of the glottic cancer patients in total, but only for early-stage cases. For cases of the hypopharynx, supraglottic larynx and nasal cavity, there was a more homogeneous result as depicted in Fig. 5.

ChatGPT 4.0 also recommended clinical trial as a third- or fourth-line therapy in some cases. Claude 3 Opus did not recommend this therapy. The age of the patients was used by ChatGPT 4.0 and Claude 3 to assess patients addressing the potential limitations of aggressive surgery or chemotherapy. The MDT where this study was performed recommended surgery for 86% of the patients (43 out of 50 patients), except for patients that had a quite advanced tumor infiltration and unresectable primary or metastasis.

Both LLMs stated that the final decision needs to be discussed with a physician and only after careful consideration of the patient’s preferences, overall health status, and potential risks and benefits of each approach, while even addressing some of the major limitation of the MDT including the fact that the prognosis of a patient can vary widely, and the difficulty to predict without knowing all the specific details of the case and the response to treatment. Claude 3 Opus and ChatGPT 4.0 listed a variety of therapy options, while ChatGPT 4.0 in general seemed to list an even larger number of therapy options. On the other hand, most of these therapy options, such as targeted therapies of primary immunotherapy are not in line with NCCN or national guidelines. Interestingly, Claude 3 and ChatGPT 4.0 were able to name different surgical approaches. A summary and depiction of the results is presented in Fig. 2. In the current version Claude 3 Opus does not list the source material of its answers, while ChatGPT 4.0 in some cases even cites recent clinical studies including a link to the manuscript.

## Discussion

This study is the first assessment of the recently published LLM Claude 3 Opus, as an auxiliary tool for the diagnostic workup and for clinical decision making in an MDT setting for HNSCC patients.

To evaluate the results of Claude 3 Opus LLM, its recommendations were compared to those provided by ChatGPT 4.0 which is currently considered the most advanced AI tool [1, 3, 4]. Using deep learning techniques, ChatGPT leverages a neural network architecture and revolutionized natural language processing (NLP) and was introduced in 2017 [18].

Claude 3 and ChatGPT are tools using Natural Language Processing (NLP), a subset of artificial intelligence (AI) dedicated to analyzing human language, a technique that has found widespread application in various medical fields. These include breast cancer research, rheumatology, medical education, and medical examinations [3, 19–23]. The diagnostic accuracy of NLPs is one of their core strengths, with studies suggesting ChatGPT to provide accurate primary diagnoses in even complex medical cases, albeit with certain limitations [24]. For example, decision making with ChatGPT has been tested for patients with primary breast cancer, in which the treatment recommendations provided by ChatGPT 3.5 or 4.0 were compared with those of the conventional MDT showing a high degree of congruence [3]. Notably, for primary breast cancer, surgery was recommended in 80% of cases. As in our study, where treatment recommendations by ChatGPT oftentimes favored surgery, this was also true in a clinical case series of laryngology and oncological cases [24]. In an alternative study approach, investigators analyzed the answers to 10 fictional cases of patients with advanced cancer with genetic alterations provided by 4 different LLMs and one physician to identify personalized treatment options. Various characteristics, including the number of treatment options, precision, recall, F1 score of LLMs were compared with human expert’s recommendation. While LLMs were able to provide large numbers of different therapeutic options, they did not provide any convincing clinical reasoning to support their recommendations [25].

Similarly, in our study ChatGPT 4.0 recommended a large number of therapeutic options for head and neck cancer treatment, while Claude 3 Opus presented a maximum of 4 different treatment options, one of them being palliative care. This behavior resembles a more realistic approach, since typically an MDT is tasked with providing one or two therapy recommendations suitable for the individual patient and not merely listing all potential options recommended. One major difference is that Claude 3 did not recommend primary immunotherapy for the patients in this study, which is in line with the current treatment guidelines for primary head and neck cancer [26–28]. ChatGPT 4.0 however did recommend immunotherapy as a primary therapeutic option for some of the patients in this study, further emphasizing that Claude 3 may be able to provide a more up to date, but also more nuanced assessment. Claude 3 did recommend induction chemotherapy with TPF (docetaxel/cisplatin/5-FU) for certain locoregionally advanced cases. This therapy is established as part of an organ preserving treatment approach in patients with laryngeal and hypopharyngeal SCCs, who are not eligible for partial laryngectomy. Another potential use is for unresectable oral cavity cancer; albeit there is currently no randomized data to support the routine use of ICT in these patients. Its use in low-risk HPV-associated oropharyngeal cancer as a de-escalation and stratification tool that is currently used in many centers, mainly in the context of ongoing studies [12, 29, 30]. Discrepancies between the two different LLMs were seen in this study especially for oropharyngeal squamous cell carcinoma and glottic laryngeal carcinoma. Due to the small number of patients in each group when performing a subsite analysis, the results of this study are limited, but a general trend can be seen that ChatGPT 4.0 prefers surgery for OPSCC, while Claude 3 Opus recommends radio(chemo)therapy. The impact of the HPV status was also analyzed briefly, but likely due to the small number of HPV-associated OPSCC in this study (8 patients), did lead to the observation that Claude 3 Opus recommends surgery for HPV-negative OPSCC. This might be in line with HPV-associated OPSCC demonstrating better survival rates compared to HPV-negative cases, leading to investigations into different treatment and de-escalation strategies to reduce or change chemoradiotherapy protocols [31]. Whether LLMs take this into account, needs to be investigated in a future study, with a larger number of HPV-positive OPSCC and additional patient characteristics. In addition, the differences between the therapy recommendations for glottic cancer need to be evaluated in larger cohorts, since this study demonstrated that Claude 3 Opus recommends radiotherapy for early-stage glottic cancer, while ChatGPT on the other hand recommends surgery for glottic cancer of any stage. Since the therapy regimen of glottic cancer is currently changing with strategies involving induction therapy and radiotherapy [12], ChatGPT’s recommendation of surgery for almost all of the cases in this study needs to be carefully evaluated. Unfortunately, the source of the information of the LLM is currently not available, with ChatGPT at least highlighting some of the studies it referred to.

Overall, ChatGPT 4.0 reached slightly higher scores for explanation, recommendation, and summarization in this study, while the biggest difference is the overall number of therapy options recommended [19, 20, 24]. The reason for this discrepancy is currently unknown since there is no function to display citations in Claude 3 Opus. Claude 3 Opus did not provide any detailed source data to support this notion, particularly regarding the regional origin of any studies. This may be a reason for using ChatGPT 4.0 in the future as a tool to inform patients to empower shared decision making, while Claude 3 Opus may be more suitable to assist the MDT. Since this is the first study investigating the use of Claude 3 Opus, there is a lack of data to back up this data or to compare the results to.

For both Claude 3 and ChatGPT 3.5, the source information on which results and recommendations are based is not provided by the AI tool [5, 6, 16]. Compared to ChatGPT 4.0 which provides guidelines and clinical studies as references, this is a major impediment when attempting to validate the answers provided by Claude 3 Opus. The results of this study are limited by a few aspects. Firstly, AI in the currently accessible form of an LLM relies on the quality and the quantity of the data used to write the prompt. Additionally, the answers of Claude 3 and ChatGPT are also formulated based on the reproduction of text. Creative thinking is therefore limited to the mere recreation of data that has been generated, which results in the lists of therapy recommendations in this study [3, 23]. These lists are reproductions of information of databases, since presently, large language models lack the capability to consider an individual patient’s personal circumstances or customize therapy guidelines accordingly. Another limitation of this study is that, although this proof-of concept study analyzes the first patient cohort and is therefore the largest study about Claude 3 Opus, only a small number of patients was examined. Patient heterogeneity may therefore influence the results of this study. Another limitation is that this monocentric study investigated the MDT of only one European institution, and the differences in therapy recommendations between Claude 3 and ChatGPT 4.0 clearly demonstrate the heterogeneity and geographical factor of different therapeutic approaches for head and neck cancer as seen in the case of a patient with early vocal fold malignancy.

Due to the novelty of Claude 3 Opus, different prompts were tested. Prompts for the diagnostic part of this study were based on the AIPI, whereas for therapy, the prompts were designed to mimic the way patients are presented to the MDT at our institution. Of note, other institutions may present patients differently, based on geographical/historical context, which further limits the universal applicability of our study results [21, 25].

Importantly, Claude 3, as ChatGPT is not programmed to think independently, but produces output based on public documents and databases [1]. It does not possess the ability to tailor individual patient treatment plans. In this study recurrent or metastatic disease was not included. Future studies therefore need to investigate this even more heterogeneous patient population. In the MDT many of these patients require thorough evaluation and more personalized treatment plans, especially when therapy guidelines are not applicable anymore and molecular tumor characteristics are discussed. Based on the results of this study, the discussion of these cases will be increasingly challenging for LLMs due to the lack of personalization and the tendency of LLMs to not ask for additional information, which is contrary to the manner of the MDT in these cases. On the other hand LLMs may benefit the MDT by summarizing the most complex clinical situations and collecting the information of hundreds of different clinical studies.

All mentioned limitations emphasize that, at this point, LLMs have to be considered an auxiliary tool rather than a replacement of the MDT [5, 6, 16]. In the future AI may assist or even validate clinical decision-making processes for HNSCC, its major advantage being the ability to incorporate the results of all available knowledge, including the most recent studies. Nonetheless it will remain the clinicians or the MDTs task to carefully evaluate the recommendations based on their clinical knowledge. Even though the performance of LLMs is increasing steadily, it is challenging to determine the day when LLMs will be able to replace/guide the clinical decision making, since randomized clinical trials are missing and are difficult to establish. An animal study, where a clinician recommends a therapy compared to a group where the therapy is recommended by an LLM, and subsequent analysis of the clinical outcome and prognosis might be one approach for this.

The superior results of Claude 3 Opus as the more recent LLM for the diagnostics of primary HNSCC in this study mark another improvement in the evolution of LLMs, whereas the therapy recommendations are similar to the results of ChatGPT 4.0. Rapid advancements in the field of AI have already started the transition of large language models (LLMs) from general language understanding to practical applications in complex medical scenarios, particularly in the context of head and neck cancer. As LLMs as seen in this study, continue to improve, we can anticipate even more advanced LLMs that may evolve beyond mere assistants to become essential guides for clinical decision-making. Nonetheless, the MDT remains essential for validating the results and ensuring the accuracy and reliability of AI-driven clinical decisions.

## Conclusions

This is the first study assessing the use of Claude 3 Opus for primary head and neck cancer, comparing its results to the answers of ChatGPT 4.0. Both LLMs demonstrated convincing results for recommending the diagnostics and therapy of primary head and neck cancer, while the answers remained general and lacked the capability to consider an individual patient’s personal circumstances or customize therapy guidelines. Claude 3 was superior in terms of diagnostic recommendations but is limited by a lack of information on the source material.
